# The gut microbiota–metabolite axis in polycystic ovary syndrome: differential characteristics between infertile and conceived populations

**DOI:** 10.3389/fmicb.2026.1705096

**Published:** 2026-03-27

**Authors:** Shen Lin, Shi-yi Qi, Dan-ting Ma, Juan Yang, Xue-li Chen, Tong Lin, Huan-huan Gao, Hui-yu Chen, Yu-nan He, Jie Lin, Jia-hui Qiu, Di Liu, Jin-bang Xu

**Affiliations:** 1 Department of Traditional Chinese Medicine, Fujian Maternity and Child Health Hospital, College of Clinical Medicine for Obstetrics and Gynecology and Pediatrics, Fujian University of Traditional Chinese Medicine, Fuzhou, Fujian, China; 2 School of Acupuncture-Moxibustion and Tuina, Fujian University of Traditional Chinese Medicine, Fuzhou, Fujian, China; 3 Computational Virology Group, Wuhan Institute of Virology, Chinese Academy of Sciences, Wuhan, Hubei, China

**Keywords:** biomarkers, gut microbiota, infertility, metabolomics, polycystic ovary syndrome

## Abstract

**Background:**

Infertility affects 70–80% of women with polycystic ovary syndrome (PCOS). However, the clinical, microbial, and metabolic factors that distinguish infertile PCOS patients from those who conceive remain poorly defined.

**Methods:**

This cross-sectional study enrolled 80 PCOS patients (35 with prior spontaneous conception [PCOS-Control] and 45 infertile [PCOS-Infertile]). Clinical characteristics, reproductive hormones, and metabolic parameters were assessed. Gut microbiota composition was analyzed by 16S rRNA sequencing, and serum metabolomic profiling was performed using UPLC-QTOF-MS. Multi-omics integration and machine learning were applied to identify discriminative features.

**Results:**

Infertile patients exhibited significantly higher testosterone levels and LH/FSH ratios. While overall gut microbial diversity was similar, taxon-specific analysis revealed enrichment of *Turicibacter* and *Prevotella* and depletion of beneficial taxa such as *Alistipes finegoldii* in the infertile group. Serum metabolomics identified seven differential metabolites, with elevated pro-inflammatory metabolites (e.g., phosphatidic acid, trichostachine) in infertile patients and reduced levels of potentially protective metabolites (e.g., L,L-Cyclo(leucylprolyl)). A multi-omics predictive model achieved strong diagnostic performance (AUC = 0.833) for identifying infertility.

**Conclusion:**

Infertility in PCOS is associated with distinct gut microbiota and serum metabolite signatures, characterized by specific microbial taxa shifts and metabolic dysregulation. These findings provide potential biomarkers for clinical stratification and offer insights into the microbiota-metabolite-fertility axis in PCOS.

## 1 Introduction

Polycystic ovary syndrome (PCOS) is a prevalent endocrine disorder affecting 6–20% of reproductive-aged women worldwide. It is characterized by hyperandrogenism, ovulatory dysfunction, and polycystic ovarian morphology (Rustam et al., 2024). Notably, infertility affects 70–80% of women with PCOS, primarily due to anovulation and metabolic disturbances such as insulin resistance (IR) and obesity (Kulkarni et al., 2023; Parua et al., 2025). Although the pathogenesis of PCOS has been extensively studied, the mechanisms underlying infertility, particularly those distinguishing women who achieve spontaneous or assisted conception from those who do not, remain incompletely elucidated.

Emerging evidence underscores the interplay between gut microbiota dysbiosis and metabolic–endocrine dysregulation in PCOS. Alterations in the gut microbiota may promote systemic inflammation, disrupt bile acid metabolism, and modulate androgen synthesis via the gut–ovary axis (Corrie et al., 2023). Specific bacterial taxa, such as *Bacteroides* and *Prevotella*, have been associated with IR and hyperandrogenemia in PCOS (Li et al., 2023; Rizk and Thackray, 2021). Moreover, serum metabolomic profiling in PCOS patients has revealed elevated triglyceride (TG) levels and reduced sex hormone-binding globulin (SHBG), both of which correlate with microbial changes and reproductive outcomes (Qi et al., 2021). However, most previous studies have focused on general PCOS populations without stratification by fertility status, leaving a critical gap in understanding how microbiota–metabolite crosstalk contributes to infertility.

Obesity and visceral adiposity are key modifiers of PCOS phenotypes. Elevated waist-to-hip ratio (WHR) and body mass index (BMI) exacerbate IR, impair ovarian folliculogenesis, and reduce oocyte quality (Snider and Wood, 2019; Yang et al., 2021). Recent studies have shown that gut microbiota composition differs significantly between obese and lean PCOS subgroups, with the Firmicutes-to-Bacteroidetes ratio correlating with homeostatic model assessment of insulin resistance (HOMA-IR) (Zhou et al., 2024; Wayadande and Honklas, 2023). Nevertheless, whether these microbial–metabolic signatures differ between PCOS women with divergent fertility outcomes remains unexplored.

This study aimed to address these gaps by comprehensively comparing clinical characteristics, serum metabolic profiles, and gut microbiota composition between the infertile and conceived PCOS groups. We hypothesized that infertile PCOS patients would exhibit distinct microbiota–metabolite interactions associated with exacerbated IR and hyperandrogenism, which may collectively contribute to impaired fertility. Our findings are expected to provide novel insights into the microbiota–metabolism–fertility axis in PCOS and inform the development of targeted therapeutic strategies.

## 2 Methods

This study enrolled 80 women aged between 20 and 40 years with PCOS who sought medical consultation at Fujian Provincial Maternity and Child Health Hospital between August 2023 and August 2024. All participants were first-visit patients who had not previously received any PCOS-specific treatment. Written informed consent was obtained from all individuals, and participants were categorized into either the PCOS-infertile group or PCOS-control group. The study protocol was approved by the Ethics Committee for Human Research of Fujian Maternity and Child Health Hospital (Approval No.2023KYLLR01032-02). Recruitment and enrollment procedures are summarized in Supplementary Figure 1.

### 2.1 Study participants

#### 2.1.1 Diagnostic criteria

PCOS was diagnosed according to the Rotterdam criteria (2003), requiring the presence of at least two of the following:

1. Oligo-ovulation and/or anovulation;
2. Clinical and/or biochemical hyperandrogenism (free androgen index, FAI ≥ 4.5);
3. Polycystic ovaries on ultrasound (≥12 follicles [2–9 mm] per ovary).

The PCOS-infertile group was defined as women who failed to conceive after ≥12 months of regular unprotected intercourse.

The PCOS-control group included women with at least one natural pregnancy within the past 2 years, all of which were spontaneous conceptions without any fertility treatments.

#### 2.1.2 Inclusion and exclusion criteria

Inclusion criteria:

1. Age 20–40 years;
2. Met the Rotterdam diagnostic criteria for PCOS;
3. Completed all study assessments.

Exclusion criteria:

1. Other endocrine disorders (e.g., thyroid dysfunction, hyperprolactinemia, Cushing syndrome, androgen-secreting tumors, diabetes, congenital adrenal hyperplasia, or 21-hydroxylase deficiency);
2. Severe systemic diseases (e.g., hepatic or renal impairment and autoimmune disorders);
3. Structural reproductive abnormalities (e.g., uterine malformations and tubal occlusion);
4. Male factor infertility, as confirmed by semen analysis;
5. Use of hormonal medications, antibiotics, or probiotics within 1 month before enrollment.

### 2.2 Data collection procedures

#### 2.2.1 Demographic and lifestyle data

Demographic information included age, height, weight, and body mass index (BMI, kg/m^2^) calculated according to World Health Organization (WHO) criteria.

Lifestyle factors assessed were smoking (yes/no; defined as ≥1 cigarette per day), alcohol consumption (yes/no; ≥1 drink per day), and coffee intake (yes/no; ≥1 cup per day).

#### 2.2.2 Clinical sample collection and analysis

All samples were collected after an 8-h fast during days 2–5 of the menstrual cycle (follicular phase).

Reproductive hormones: A 5 mL of venous blood sample was collected, and serum was separated by centrifugation (3,000 rpm for 10 min). Serum levels of estrogen (E_2_), luteinizing hormone (LH), follicle-stimulating hormone (FSH), prolactin (PRL), and total testosterone (T) were measured using an automated chemiluminescence immunoassay system.

Glucose tolerance test (OGTT) and insulin assay: Fasting blood samples (0 h) were collected, followed by samples at 30 min, 1 h, 2 h, and 3 h after a glucose load. Serum insulin concentrations were measured using an electrochemiluminescence immunoassay (ECLIA) on a Cobas e601 analyzer. Insulin resistance was evaluated using the HOMA-IR: (FPG [mmol/L] × FINS [μU/mL]) / 22.4 (Rhaiem et al., 2025).

Biochemical indicators: Fasting serum levels of alanine aminotransferase (ALT), blood urea nitrogen (BUN), creatinine (Cr), triglycerides (TG), total cholesterol (TC), low-density lipoprotein cholesterol (LDL-C), and high-density lipoprotein cholesterol (HDL-C) were measured using an enzymatic colorimetric method on an automated biochemical analyzer. All assays were performed following the manufacturer’s protocols with routine quality control procedures in the central clinical laboratory of the hospital.

### 2.3 Gut microbiota analysis

Fecal sample collection: All participants, including those in the PCOS-control group, provided fresh fecal samples before pregnancy. Samples were collected during days 2–5 of the spontaneous menstrual cycle (follicular phase) to control for hormonal fluctuations. Fecal samples were stored at −80 °C within 2 h of collection.

#### 2.3.1 16S rRNA sequencing

DNA was extracted using the QIAamp Fast DNA Stool Mini Kit. The V3–V4 regions were amplified (primers 341F: 5′-CCTACGGGNGGCWGCAG-3′; 806R: 5′-GGACTACHVGGGTATCTAAT-3′) and sequenced on an Illumina NovaSeq 6,000 (2 × 250 bp).

Bioinformatics: Raw sequencing reads were processed in QIIME2. Sequences were denoised using DADA2, taxonomically classified against the SILVA v138.1 database, and analyzed for *α* diversity (Shannon index) and *β* diversity (Bray–Curtis distance).

### 2.4 Serum metabolomics analysis

Sample preparation: Fasting venous blood was collected (8–10 a.m.), centrifuged (3,000 rpm, 10 min, 4 °C), and the resulting serum was stored at −80 °C. For metabolomic analysis, 100 μL of serum was mixed with 400 μL of ice-cold methanol:acetonitrile (1,1, v/v) for protein precipitation. After vortexing, incubation (−20 °C, 30 min), and centrifugation (14,000 rpm, 15 min), the supernatant was dried under a nitrogen stream and reconstituted in 50% acetonitrile.

LC–MS analysis: Analysis was performed using a UPLC-QTOF-MS (Waters Xevo G2-XS) equipped with an ACQUITY UPLC HSS T3 column (2.1 mm × 100 mm, 1.8 μm). The mobile phase consisted of 0.1% formic acid in water (A) and acetonitrile (B), with a gradient elution from 5 to 95% B over 20 min. MS detection was conducted in both ESI + and ESI- modes, with a scan range of m/z 50–1,200. Capillary voltages were set to 2.5 kV (ESI+) and 2.0 kV (ESI−).

Data processing: Raw data were processed using Progenesis QI for peak alignment and normalization. Metabolites were annotated using the Human Metabolome Database (HMDB) and METLIN databases. Multivariate statistical analysis, including principal component analysis (PCA) and orthogonal projections to latent structures-discriminant analysis (OPLS-DA), was performed using SIMCA 14.1.

### 2.5 Statistical analysis

#### 2.5.1 Clinical and metabolic comparisons

Continuous variables are presented as mean ± standard deviation or median (interquartile range). Group differences were assessed using the Student’s t-test (for normally distributed data) or the Mann–Whitney U-test (for non-normally distributed data). Categorical variables were compared using the chi-square test. Normality was evaluated using the Shapiro–Wilk test.

#### 2.5.2 Multi-omics integration

Differential microbial abundance was identified with LEfSe (LDA score >3.0, *p* < 0.05). Significant metabolites were selected based on OPLS-DA variable importance in projection (VIP) scores >1.0 and FDR-adjusted *p*-values <0.05, and were subsequently mapped to KEGG or PICRUSt2 pathways. Multi-omics integration was performed using sparse canonical correlation analysis (via the mixOmics R package), supported by Mantel tests for matrix correlation assessment. Spearman rank correlations were calculated and visualized in Cytoscape 3.9 to reveal clinical–metabolic–microbial associations. PICRUSt2 was used to infer metagenomic functions from 16S rRNA sequencing data, with a focus on KEGG and MetaCyc pathways. It should be noted that these are computational predictions and have inherent limitations compared with direct metagenomic sequencing or transcriptomic analysis. All analyses were conducted using R 4.3.1 (with the stats, vegan, and ggplot2 packages) and GraphPad Prism 9.0. A Random Forest classification model was built using scikit-learn 1.2 with 10-fold cross-validation to identify features predictive of infertility. Metabolic pathway enrichment analysis was performed in MetaboAnalyst 5.0 using the KEGG and HMDB databases. Statistical significance was defined as a *p*-value of < 0.05 throughout the study.

## 3 Results

### 3.1 Clinical and demographic characteristics

A total of 80 patients with PCOS were enrolled, including 35 in the PCOS-control group and 45 in the PCOS-infertile group. No significant differences were observed in baseline demographic characteristics and lifestyle factors between the two groups (Table 1).

**Table 1:** Baseline characteristics of study cohorts.

Characteristic	PCOS-control (*n* = 35)	PCOS-infertile (*n* = 45)	*p*-value
Age, years	29.49 (3.57)	30.91 (3.20)	0.068
Height, cm	159.7 (4.72)	159.6 (6.04)	0.935
Weight, kg	58.89 (9.22)	62.73 (13.67)	0.174
BMI, kg/m^2^	23.11 (3.50)	24.58 (4.72)	0.143
Smoking, *n* (%)	0 (0%)	2 (4.44%)	0.207
Alcohol consumption, *n* (%)	1 (2.86%)	4 (8.89%)	0.269
Coffee intake, *n* (%)	2 (5.71%)	3 (6.67%)	0.861

Data are presented as mean (standard deviation) for continuous variables and as number (percentage) for categorical variables.

### 3.2 Hormonal and metabolic profiles

The PCOS-infertile group exhibited significantly higher levels of testosterone and LH/FSH ratio compared with the PCOS-control group. No significant differences were observed in FSH, LH, PRL, or E_2_ between the two groups. Although none of the individual time points in the OGTT or insulin assay were statistically significant, median values for both OGTT and insulin were consistently higher in the PCOS-infertile group. Additionally, serum Cr levels were significantly higher in the PCOS-infertile group than in the PCOS-control group (Table 2).

**Table 2:** Reproductive hormones, glucose metabolism, insulin resistance, and biochemical indicators of study cohorts.

Parameter	PCOS-control (*n* = 35)	PCOS-infertile (*n* = 45)	*p*-value
Reproductive hormones			
FSH	5.800 (1.367)	5.051 (1.655)	0.060
LH	5.402 (3.149)	6.432 (3.917)	0.265
LH/FSH ratio	0.931 (0.487)	1.298 (0.597)	**0.011**
Testosterone	0.393 (0.150)	0.525 (0.143)	**0.0004**
PRL	15.44 (12.76)	14.98 (7.46)	0.848
Estradiol	30.09 (19.90)	35.22 (15.09)	0.241
Oral glucose tolerance test			
0 h	5.159 (0.513)	5.418 (1.182)	0.254
1 h	8.529 (2.411)	9.456 (4.081)	0.291
2 h	6.989 (1.970)	8.128 (4.192)	0.176
3 h	5.549 (1.939)	5.957 (2.859)	0.533
Insulin assay			
0 h	9.787 (5.744)	11.83 (5.857)	0.100
1 h	99.75 (64.33)	96.91 (57.33)	0.853
2 h	88.83 (70.05)	105.0 (80.96)	0.391
3 h,	42.58 (48.43)	53.90 (63.79)	0.452
HOMA-IR	2.229 (1.376)	2.944 (1.719)	0.061
Biochemical indicator			
ALT	16.49 (11.39)	23.02 (28.98)	0.337
BUN	4.330 (0.89)	4.017 (1.15)	0.347
Cr	56.15 (10.96)	62.86 (7.28)	**0.018**
TC	4.875 (0.645)	4.939 (0.921)	0.781
TG	1.449 (1.013)	1.377 (0.633)	0.764
HDL-C	1.262 (0.311)	1.220 (0.235)	0.602
LDL-C	2.662 (0.356)	2.778 (0.670)	0.457

Data are presented as mean (standard deviation). Bold p-values indicate statistical significance (*p* < 0.05).

### 3.3 Gut microbiota alterations

#### 3.3.1 Diversity and structure

**Figure 1:**
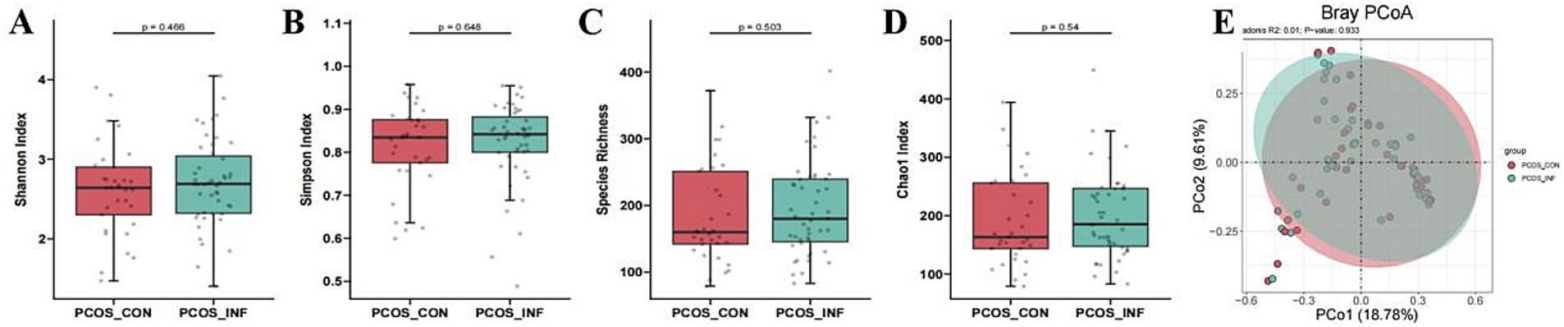
Alpha diversity and beta diversity analyses of the gut microbiota among the different groups based on the 16S rRNA gene sequences **(A–D)** Shannon index **(A)**, Simpson index **(B)**, species richness **(C)**, Chao1 index **(D)**, and PCoA analysis of intestinal microflora **(E)**.

16S rRNA sequencing data from 80 samples revealed no significant differences in *α* diversity or β diversity between the PCOS-control and PCOS-infertile groups, indicating that the microbial community structure was similar regardless of fertility status (Figure 1).

Specifically, the Shannon index (2.617 ± 0.574 vs. 2.703 ± 0.544, *p* = 0.466), Simpson index (0.814 ± 0.095 vs. 0.822 ± 0.096, *p* = 0.648), species richness (184.39 ± 73.48 vs. 193.34 ± 69.55, *p* = 0.503), and Chao1 index (191.41 ± 78.72 vs. 201.00 ± 74.86, *p* = 0.540) did not differ significantly between groups. For β diversity: Bray–Curtis PCoA analysis showed Adonis results of PCo1 = 18.78%, PCo2 = 9.61%; R^2^ = 0.01; *p* = 0.933 (Figure 1).

#### 3.3.2 Taxon-specific alterations

**Figure 2:**
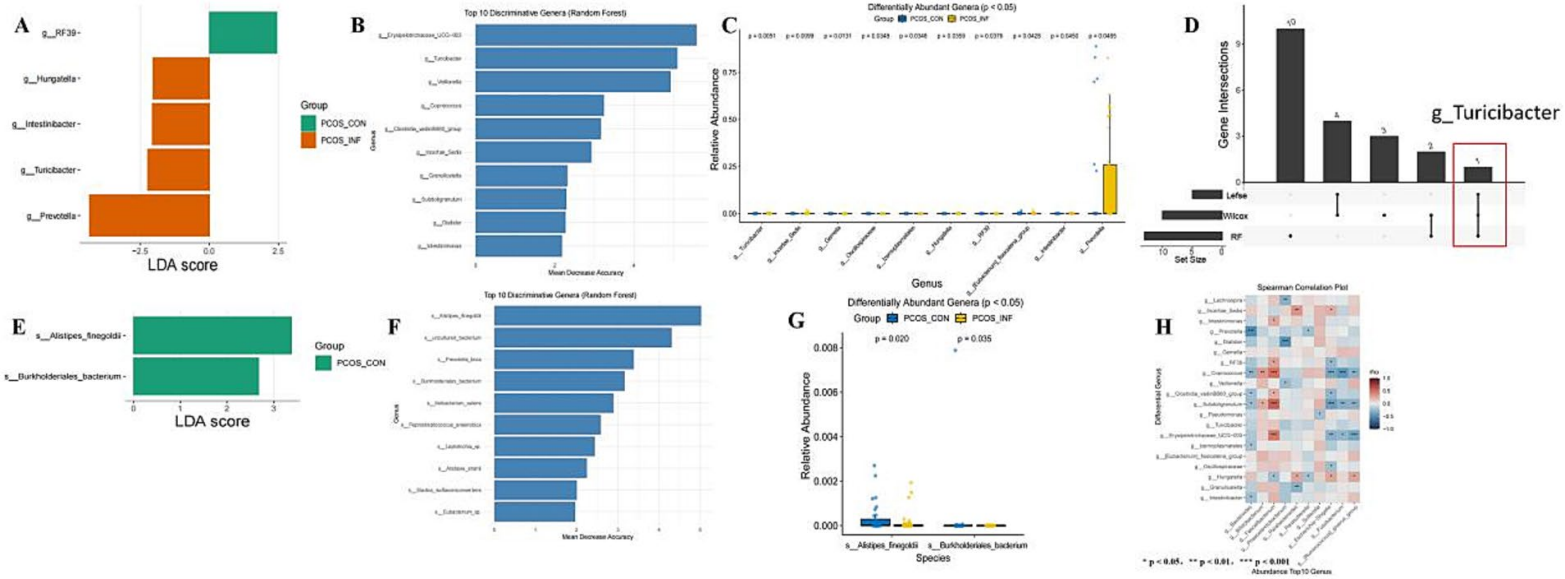
Differences in gut microbiota between PCOS-infertile and PCOS-control groups. **(A–D)** Differential taxa identified by LEfSe, RF, and the Wilcoxon test at the genus level. **(E,F)** Functional biomarkers identified by LEfSe, RF, and Wilcoxon tests at the species level. **(H)** Heatmap shows correlation analysis between differential bacteria and the top 10 most abundant genera.

LEfSe analysis identified several discriminative taxa (LDA > 2.0). A combined approach using LEfSe, Random Forest (RF), and the Wilcoxon test identified 20 differential bacterial genera. Among these, *Turicibacter* was the only genus consistently detected by all three methods (Figure 2). At the species level, eight species were identified, with *Alistipes finegoldii* and *Burkholderiales bacteria* highlighted by all three analytical approaches (Figure 2).

Genera enriched in the PCOS-infertile group included *Prevotella* (LDA = 4.34, *p* = 0.048), *Turicibacter* (LDA = 2.23, *p* = 0.005), *Intestinibacter* (LDA = 2.06, *p* = 0.043), and *Hungatella* (LDA = 2.04, *p* = 0.033). In contrast RF39 (LDA = 2.44, *p* = 0.041) and *Gemella* (LDA = 1.042, *p* = 0.011) were depleted in the infertile group. At the species level, *Alistipes finegoldii* (LDA = 3.38, p = 0.043) and *Burkholderiales bacterium* (LDA = 2.67, *p* = 0.033) were also reduced in the PCOS-infertile group.

#### 3.3.3 Correlation analysis between different bacteria and top 10 bacterial flora

Spearman correlation analysis revealed significant interactions between the 20 differential bacterial genera and the top 10 most abundant genera (Figure 2H). *Coprococcus*, *Subdoligranulum*, *Erysipelotrichaceae*_UCG-003, and *Faecalibacterium* showed significant positive correlations (*r* = 0.497, 0.545, and 0.450, respectively; *p* < 0.001). In contrast, *Prevotella* was strongly negatively correlated with *Bacteroides* (*r* = −0.51, *p* < 0.001).

### 3.4 Serum metabolomics profiling

#### 3.4.1 Differential metabolites and multivariate analysis

**Figure 3:**
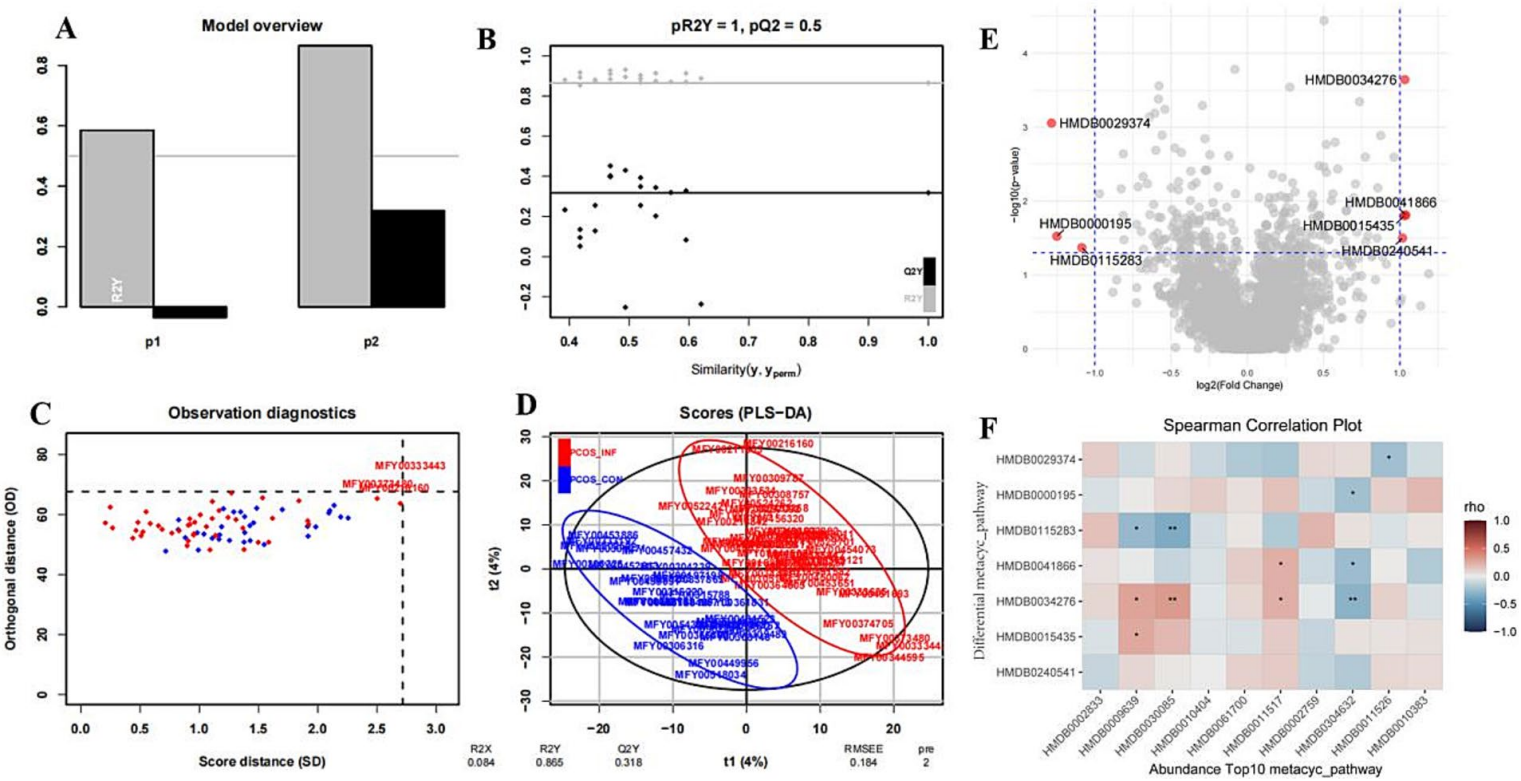
OPLS-DA score plot demonstrates distinct serum metabolic profiles between the PCOS-infertile and PCOS-control groups. **(A)** Inertia bar plot. **(B)** Significance diagnostic: pR2Y and pQ2 of the model are compared with the corresponding values obtained after random permutation of the Y response. **(C)** OPLS-DA model observation diagnostic plot. **(D)** X-score plot: The number of components and the cumulative R2X, R2Y, and Q2Y are indicated below the plot. R2Y: Fraction of the variation of the Y variables explained by the model. R2X: Fraction of the variation of the X variables explained by the model. Q2Y: Fraction of the variation of the Y variables predicted by the model. RMSEE: Root mean square error of estimation. **(E)** Volcano plot of serum metabolites. **(F)** Heatmap shows the correlation analysis between differential metabolites and the top 10 metabolites.

Untargeted metabolomics identified significant differences in serum metabolites between the PCOS-infertile and PCOS-control groups. Using thresholds of VIP ≥ 1, *p* < 0.05, and log_2_FC > 1, seven key metabolites were selected: trichostachine (HMDB0029374), inosine (HMDB0000195), phosphatidic acid (22:1/20:3) (HMDB0115283), L, L-cyclo(leucylprolyl) (HMDB0034276), deltamethrin (HMDB0041866), sodium polystyrene sulfonate (HMDB0015435), and luteolin 7-glucuronide (HMDB0240541). OPLS-DA model analysis confirmed a clear separation between the two groups (pR^2^Y = 1, pQ^2^ = 0.5) (Figure 3).

Metabolites elevated in the PCOS-infertile group included trichostachine (VIP = 2.11, log_2_FC = −1.28, *p* = 0.0009), inosine (VIP = 2.37, log_2_FC = −1.25, *p* = 0.030), phosphatidic acid (22:1/20:3) (VIP = 1.84, log_2_FC = −1.09, *p* = 0.043), deltamethrin (VIP = 1.89, log_2_FC = 1.04, *p* = 0.015), and polystyrene sulfonate (VIP = 1.92, log_2_FC = 1.03, *p* = 0.016). Metabolites reduced in the PCOS-infertile group included L, L-cyclo(leucylprolyl) (VIP = 2.93, log_2_FC = 1.033, *p* = 0.0002) and luteolin 7-glucuronide (VIP = 1.52, log_2_FC = 1.02, *p* = 0.032).

#### 3.4.2 Correlation analysis between differential serum metabolites and top10 metabolites

Correlation analysis showed that the seven differential HMDB metabolites were significantly associated with several of the top 10 most abundant metabolites (Figure 3F). HMDB0115283 was negatively correlated with HMDB0030085 (pubescenol; *r* = −0.35, *p* = 0.0014), while HMDB0034276 was positively correlated with HMDB0030085 (*r* = 0.32, *p* = 0.004).

#### 3.4.3 Pathway enrichment

**Figure 4:**
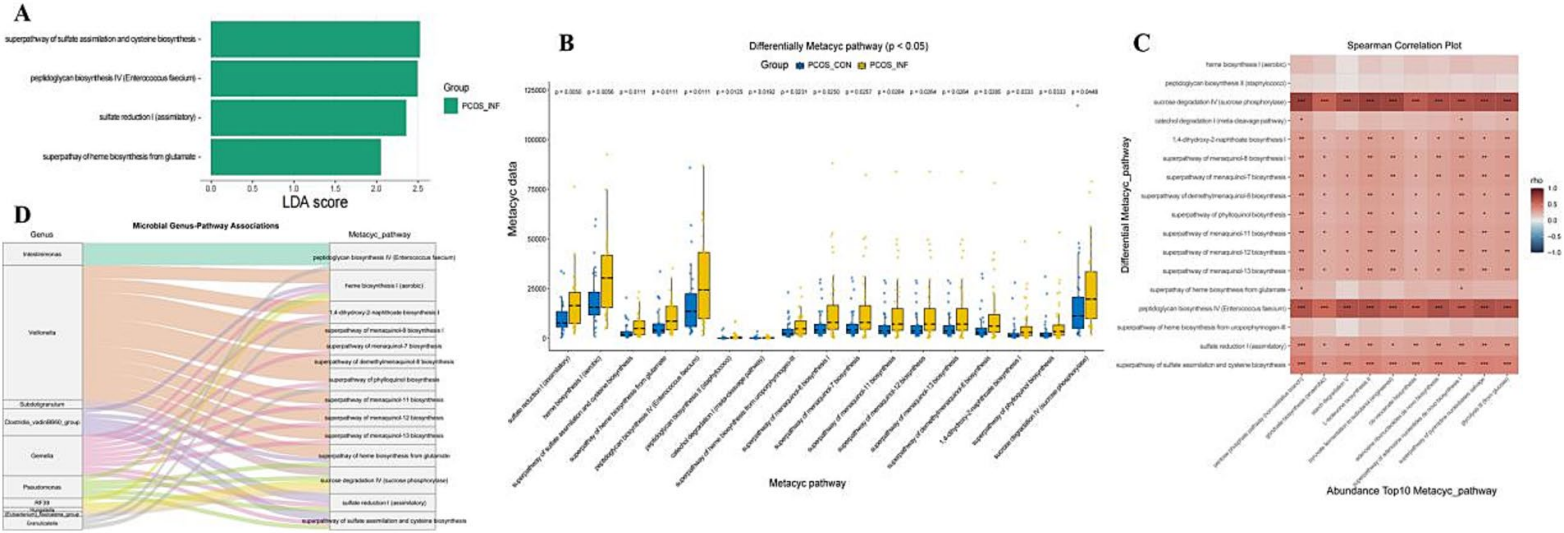
MetaCyc metabolic pathway predicted by PICRUSt2. **(A–B)** Differential pathways identified by LEfSe and Wilcoxon analyses between the PCOS-infertile and PCOS-control groups. **(C)** Heatmap shows the correlation analysis between differential metabolic pathways and the top 10 metabolic pathways. **(D)** Integrated network linking differential genera with MetaCyc pathways.

PICRUSt2-based functional prediction, combined with LEfSe and Wilcoxon analyses, identified 4 and 17 differential metabolic pathways in the PCOS-infertile and PCOS-control groups, respectively, with 4 pathways overlapping between the 2 methods. No KEGG pathways were significantly enriched (Figure 4).

Pathways significantly enriched in the PCOS-infertile group included the superpathway of sulfate assimilation and cysteine biosynthesis (LDA = 2.52, *p* = 0.016), peptidoglycan biosynthesis IV (*Enterococcus faecium*) (LDA = 2.49, *p* = 0.0068), sulfate reduction I (assimilatory) (LDA = 2.35, *p* = 0.015), and the superpathway of heme biosynthesis from glutamate (LDA = 2.05, *p* = 0.023).

Among the 20 differential microbial genera, 10 showed correlations with 14 of the 17 differential MetaCyc pathways, primarily involving *Veillonella* and *Gemella* (Figure 4D).

#### 3.4.4 Correlation analysis between the differential metacyclic metabolic pathway and top10 pathways

Most of the 17 differential MetaCyc pathways were positively correlated with the top 10 most abundant pathways (Figure 4C). Sucrose degradation IV (sucrose phosphorylase) and peptidoglycan biosynthesis IV (*Enterococcus faecium*) showed particularly strong positive correlations with highly abundant pathways.

### 3.5 Microbial–metabolite–clinical correlation analysis

#### 3.5.1 Metabolite–microbiota correlations

**Figure 5:**
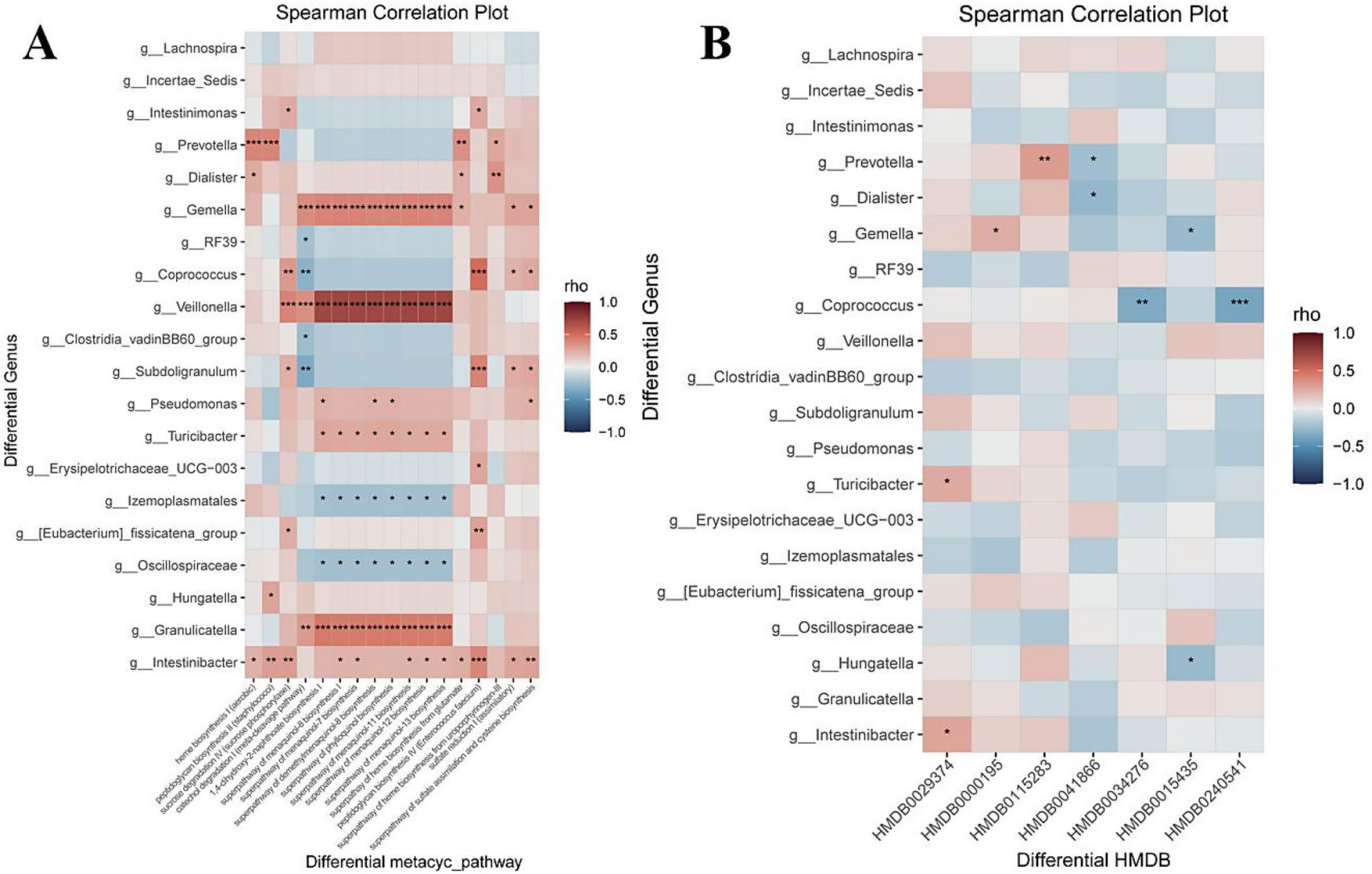
Microbial interaction networks with metabolites and metabolic pathways. **(A)** Heatmap shows the correlation analysis between differential microbes and differential MetaCyc pathways. **(B)** Heatmap shows the correlation analysis between differential microbes and differential metabolites.

*Gemella* abundance was positively correlated with serum inosine (HMDB0000195; *r* = 0.24, *p* = 0.03) and negatively correlated with polystyrene sulfonate (HMDB0015435; *r* = −0.26, *p* = 0.018). *Turicibacter* abundance was positively correlated with serum trichostachine (HMDB0029374; *r* = 0.26, p = 0.023). *Prevotella* abundance was positively correlated with serum phosphatidic acid (HMDB0115283; r = 0.31, *p* = 0.0047) and negatively correlated with deltamethrin (HMDB0041866; *r* = −0.24, *p* = 0.032) (Figure 5).

#### 3.5.2 Clinical–metabolite associations

**Figure 6:**
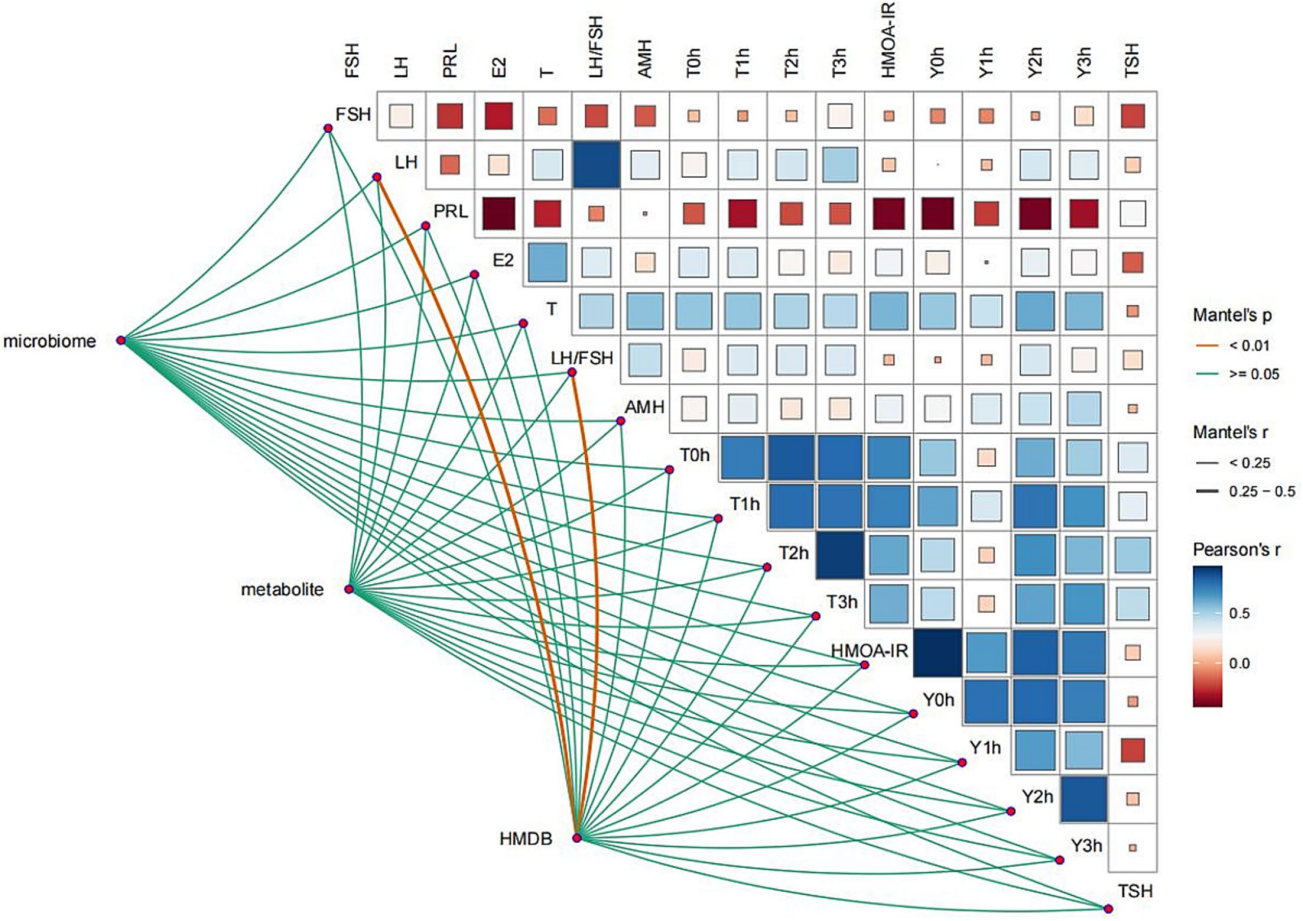
A complex Pearson’s correlation matrix illustrates clinical indicators and microbiome–metabolite–HMDB associations. To construct the correlation matrix, clinical indicators (heatmap) and differential biomarkers (lower links) were log_2_-transformed. A box inside the heatmap indicates a correlation. Blue and red represent Pearson’s *r* value, respectively. Links with *p*-values and |*r*| values show the correlation between clinical indicators and differential biomarkers. The orange and green colors indicate Mantel’s *p*-values, respectively.

Correlations between the microbiome, metabolome, or metabolic pathways and glucose tolerance or insulin measures at 0, 1, 2, and 3 h were weak (*r* < 0.25) and non-significant. In contrast, the metabolome showed a significant positive correlation with serum LH (*r* = 0.30, *p* = 0.004) and the LH/FSH ratio (*r* = 0.26, *p* = 0.008). Phosphatidic acid was significantly positively correlated with LH/FSH (*r* = 0.476, *p* = 0.034) (Figure 6).

#### 3.5.3 Predictive modeling

**Figure 7:**
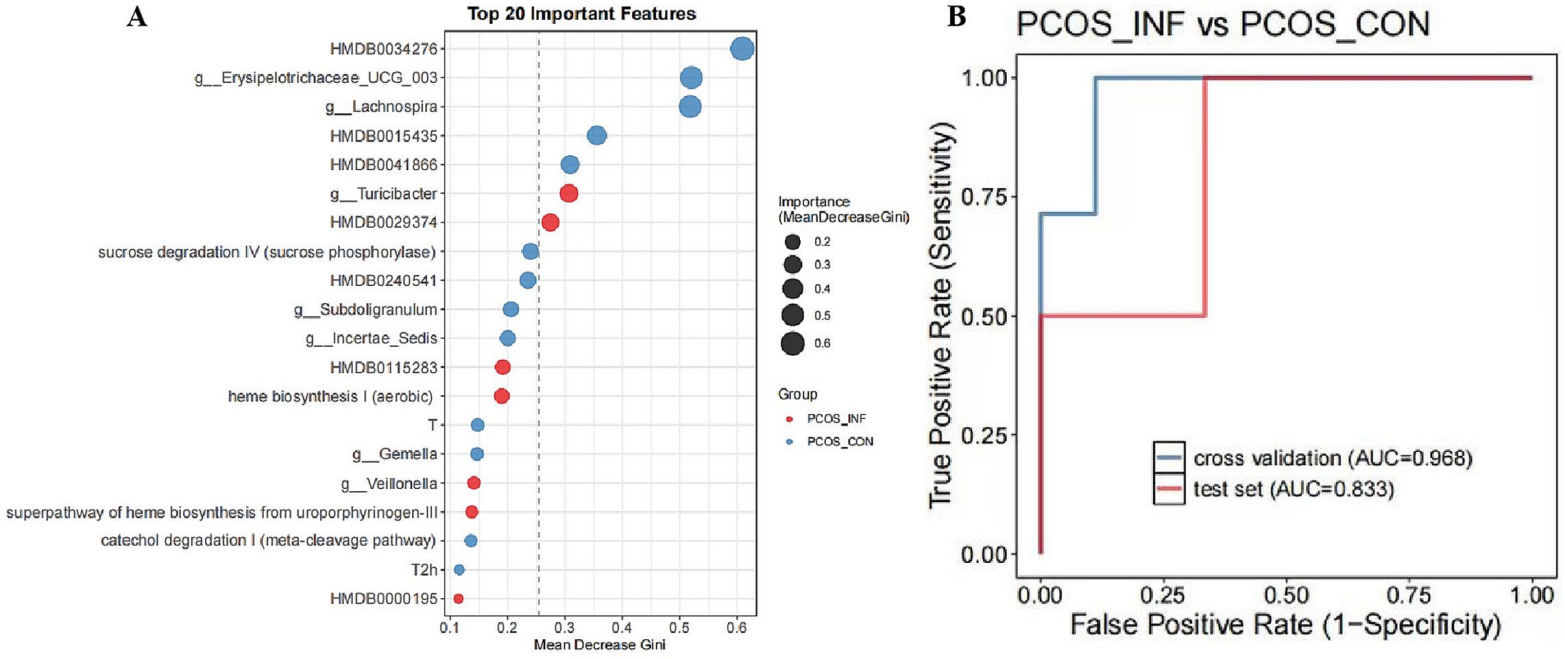
Machine learning model for PCOS classification using multi-omics features. **(A)** Top 20 important variables. The *y*-axis represents each variable, and the *x*-axis represents the mean decrease in Gini value. The larger the value, the more important the variable is. **(B)** ROC curves illustrate the classifier’s performance on the cross-validation set (AUC = 0.968) and the independent test set (AUC = 0.833).

The top 20 most important features in the predictive model included 7 microbial species, 2 clinical indicators, 7 serum metabolites, and 4 metabolic pathways. A logistic regression model built using these selected variables showed good discriminatory performance, with an area under the curve (AUC) of 0.833 on the independent test set (Figure 7). It is noteworthy that the model performed well on the independent test set (AUC = 0.833), which is a more robust indicator of generalizability than the cross-validation performance (AUC = 0.968).

Based on the mean decrease in Gini, HMDB0034276, *Erysipelotrichaceae_UCG_003*, and *Lachnospira* were more significant in the PCOS-control group, whereas *Turicibacter* and HMDB0029374 (trichostachine) were more prominent in the PCOS-infertile group. Random forest analysis identified *Erysipelotrichaceae_UCG_003* (odds ratio [OR] = 4.47, 95% confidence interval [CI]: 0.41–75.90) and *Lachnospira* (OR = 4.47, 95% CI: 0.41–75.90) as the most discriminative features for PCOS-control phenotypes (MeanDecreaseGini > 0.5), although these associations were not statistically significant (*p* = 0.315).

The metabolite HMDB0034276 showed the strongest effect size in the PCOS-control group (OR = 16.20, 95% CI: 1.09–1088.44, *p* = 0.041).

HMDB0034276, putatively annotated as a lipid mediator, exhibited pro-inflammatory association patterns. Sulfate assimilation pathways were coordinately downregulated in the PCOS-infertile group (OR = 0.62–1.61).

## 4 Discussion

This study provides the first multi-omics evidence linking gut microbial dysbiosis, serum metabolic perturbations, and endocrine dysfunction to infertility in PCOS. Our key findings reveal that, despite comparable baseline demographics, infertile PCOS patients exhibit distinct microbial–metabolic–endocrine signatures associated with reproductive dysfunction, independent of classical obesity or insulin resistance phenotypes.

### 4.1 Endocrine dysregulation as the core phenotype

Our study reaffirms that hyperandrogenemia and pulsatile dysregulation of gonadotropin-releasing hormone are central to PCOS-related infertility. We observed significantly higher testosterone and LH/FSH ratios in infertile PCOS patients, consistent with previous reports. Elevated free testosterone is observed in approximately 80–90% of individuals with PCOS (Azziz et al., 2019).

An increased LH/FSH ratio is a well-recognized marker of infertility in PCOS, as it disrupts normal follicular development and can lead to ovulatory dysfunction. Specifically, suppressed FSH delays follicular maturation, while elevated LH stimulates excessive testosterone production by ovarian stromal cells (Lei et al., 2024).

The elevated LH/FSH ratio and FAI levels in infertile PCOS women further support the concept that hyperandrogenism exacerbates anovulation, potentially through direct inhibition of aromatase activity in granulosa cells (Al-Jefout et al., 2017). These findings align with the Rotterdam consensus guidelines, which emphasize the importance of metabolic screening in the management of PCOS-related infertility.

### 4.2 Distinct microbial signatures differentiate fertile and infertile PCOS patients

In contrast to studies comparing PCOS patients with healthy controls (Yu et al., 2022). Our study specifically compares PCOS women with and without a history of conception.

We found that *α* diversity did not differ between groups, suggesting that functional alterations in specific microbial taxa–rather than overall diversity—underlie the pathogenesis of infertility. Despite the lack of overall structural differences observed in α- or β-diversity between groups, indicating similar overall community structure, subsequent taxon-specific analyses revealed distinct microbial compositions at the genus and species levels.

The combination of LEfSe, Random Forest, and Wilcoxon test analyses robustly identified specific taxa altered in infertile PCOS, underscoring that compositional shifts in key microbes may influence reproductive outcomes.

We found that *Turicibacter* was significantly enriched in the gut of infertile PCOS patients. Elevated *Turicibacter* abundance has been linked to intestinal inflammation in mice and may exacerbate metabolic endotoxemia, potentially contributing to systemic inflammation in infertile PCOS (Wang et al., 2021).

In sheep, *Turicibacter* abundance was higher in non-pregnant than in pregnant ewes (Ding Hongxiang et al., 2022), suggesting that it may serve as a microbial indicator of reproductive dysfunction risk. *Turicibacter* species can modulate host bile acid and lipid metabolism (Lynch et al., 2023) and have been negatively correlated with taurine deoxycholic acid (TUDCA). Notably, fecal microbiota transplantation from PCOS patients into mice resulted in PCOS-like phenotypes and significantly reduced TUDCA levels (Qi et al., 2019).

Conversely, the abundance of *Alistipes finegoldii* and *Burkholderiales* bacteria was reduced in infertile PCOS patients. These bacteria produce short-chain fatty acids (SCFAs), which help maintain intestinal health and immune homeostasis.

SCFAs promote mucin production by intestinal epithelial cells, enhance barrier function, and suppress the production of pro-inflammatory cytokines (Aleti et al., 2023). By activating G-protein-coupled receptors, inhibiting HDAC, and modulating cytokine secretion, SCFAs help regulate immune and barrier functions, thereby maintaining intestinal immune homeostasis (Dalile et al., 2019).

A reduction in these SCFA-producing bacteria may compromise intestinal barrier integrity and immune regulation, increasing susceptibility to inflammatory conditions (Goncalves et al., 2018). *Alistipes finegoldii*, in particular, contributes to host energy metabolism, anti-inflammatory responses, and intestinal barrier maintenance through SCFA production (Ceperuelo-Mallafre et al., 2025).

The strong positive correlation between *Faecalibacterium* (a butyrate producer) and genera such as *Coprococcus* and *Subdoligranulum* suggests the presence of cross-feeding relationships that support metabolic stability (Morris et al., 2013). Such interactions help maintain the metabolic balance, facilitate butyrate production, and exert anti-inflammatory, barrier-strengthening, and immunomodulatory effects (Martin et al., 2023). In contrast, the negative correlation between *Prevotella* and *Bacteroides* likely reflects competition for mucosal niches.

### 4.3 Dysregulated serum metabolites reflect altered microbial–host co-metabolism and multiple pathological axes in PCOS-infertile patients

Serum metabolomics analysis identified seven differentially abundant metabolites in infertile PCOS patients, pointing to three interconnected pathological axes: microbial–host co-metabolism, chronic inflammation, and xenobiotic stress.

Several elevated metabolites appeared to act as pro-inflammatory and metabolic stress drivers. Phosphatidic acid (22:1/20:3) (HMDB0115283) and inosine (HMDB0000195) were among the top 20 most discriminative features. An abnormal lipid profile are among the metabolic characteristics of PCOS. Phosphatidic acid is a key intermediate in the synthesis of phospholipids and triglycerides in cell membranes and is involved in diverse pathophysiological processes, including inflammation, proliferation, and oncogenesis. Elevated levels of multiple phospholipids and glycerophospholipid metabolites in the serum of PCOS patients suggest potential hyperactivity of the glycerophospholipid/fatty acid synthesis pathway (Yu et al., 2022). Inhibition of its synthesis has been shown to reduce diet-induced obesity and hepatic steatosis (Huang et al., 2022).

Inosine, a purine nucleoside, functions as an intermediate between purine metabolism and the adenosine cycle. A non-targeted metabolomics study revealed significantly lower serum inosine levels in patients with PCOS compared to healthy controls (Yu et al., 2021). Inosine exhibits anti-inflammatory properties by inhibiting pro-inflammatory cytokine production (Wang et al., 2023), though it may also stimulate mast cell degranulation, suggesting a context-dependent role (Ledecký et al., 2016). Moreover, inosine levels increase under conditions of cellular metabolic stress, such as inflammation (Paunescu, 2021). The elevated serum inosine levels in this study may represent a compensatory response to chronic inflammation and tissue stress.

Other notable metabolites were detected in the serum of infertile PCOS patients. Deltamethrin, a widely used insecticide, can enter the human body through the food chain or water and has been documented to be neurotoxic. Chronic exposure to fish disrupts intestinal goblet cells, promotes inflammation, and induces gut microbiota dysbiosis (Wu et al., 2022). Deltamethrin can affect ovulation, leading to follicular atresia, reduced number of follicular cells, oocytes, and corpora lutea, and inducing atrophy of endometrial glands (Marettova et al., 2017). In mice, deltamethrin exposure damages the testicular structure, leading to germ cell loss and cytoplasmic vacuolization (Ben Slima et al., 2017).

Trichostachine (HMDB0029374) emerged as the most important serum metabolite in infertile PCOS. Although human studies are limited, trichostachine, a derivative of exogenous peppericin, has been identified as a highly sensitive and specific biomarker that distinguishes inflammatory bowel disease patients from healthy individuals (Xu et al., 2022).

Polystyrene sulfonate, as a component of microplastics or drugs, may have endocrine-disrupting effects. Studies in mice have shown that exposure to polystyrene microplastics can trigger PCOS-like phenotypes, including ovarian polycystic changes and ovulatory disorders, accompanied by gonadotropin dysregulation and increased apoptosis of ovarian theca cells (Zhang et al., 2025). It is important to note that the identification of these xenobiotics via untargeted metabolomics may result from environmental exposure, background contamination, or annotation uncertainty. Targeted quantification is needed to confirm their physiological relevance.

In the PCOS-control group, L, L-cyclo(leucylprolyl) and luteolin 7-glucuronide were elevated, suggesting a possible loss of protective or metabolic-modulating factors in infertile patients.

L, L-cyclo(leucylprolyl), a small signaling molecule synthesized by gut microbes, may contribute to anti-inflammatory and antioxidant activity within the intestinal microecosystem. The functional significance of L, L-cyclo(leucylprolyl) remains unclear, with some studies linking it to increased hepatocellular carcinoma risks (Stepien et al., 2021) and others suggesting a protective role against cellular stress (Siddiqui et al., 2023). When patients with PCOS infertility lack these metabolites, they may be more prone to chronic inflammation and oxidative damage. In our study, this metabolite showed the strongest effect size among protective factors.

Luteolin 7-glucuronide, found in various fruits and vegetables, exhibits potent antioxidant and anti-inflammatory properties. Studies have shown that luteolin 7-glucuronide can inhibit the production of pro-inflammatory factors such as TNF-*α*, IL-6, and IL-1β (Seelinger et al., 2008). Furthermore, luteolin 7-glucuronide can enhance ovarian function through estrogen-like effects. Animal studies have shown that luteolin interventions benefit PCOS by improving ovulation disorders, insulin resistance, and hormonal imbalances (Huang and Zhang, 2021). While its function in PCOS infertility remains unclear and the literature is limited, this association suggests it may represent a potential protective metabolite.

Pathway enrichment analysis predicted increased activity in the superpathway of sulfate assimilation and cysteine biosynthesis, peptidoglycan biosynthesis IV (*Enterococcus faecium*), sulfate reduction I (assimilatory), and the superpathway of heme biosynthesis from glutamate in infertile PCOS patients. These inferred pathways suggest a possible link between bacterial oxidative stress responses and cell wall remodeling, although direct experimental validation is required. Enrichment of sulfate assimilation and heme biosynthesis pathways may indicate microbial adaptation to—or contribution to—a host oxidative stress environment. For instance, sulfate assimilation supports the synthesis of antioxidants such as glutathione (de Bont et al., 2022), and its upregulation may reflect a compensatory response to systemic oxidative stress, which is commonly elevated in PCOS.

Heme biosynthesis I (aerobic) and the superpathway of heme biosynthesis from uroporphyrinogen III were also among the top 20 model features in infertile PCOS. Heme synthesis is critical for host energy metabolism, inflammation, and oxidative stress regulation, and gut microbial heme metabolism may influence host–microbe interactions relevant to PCOS-related infertility (Medlock and Dailey, 2022).

### 4.4 Gut microbiota–serum metabolite crosstalk modulates reproductive outcomes

Our multi-omics integration identified several robust correlations between differential gut microbes and serum metabolites, suggesting a potential microbiota–metabolite axis contributing to PCOS infertility.

Reduced bile acid levels have been shown to induce PCOS-like phenotypes in mice (Qi et al., 2019). We observed elevated *Turicibacter* abundance in infertile women. *Turicibacter* can influence bile acid metabolism and regulate the host lipid synthesis and catabolism pathways, which may indirectly affect lipid metabolism (Lynch et al., 2023). Elevated serum PA levels in PCOS patients suggest hyperactivity of the glycerophospholipid/fatty acid synthesis pathway (Yu et al., 2022). While no evidence has demonstrated that *Turicibacter* directly produces specific phospholipid acid molecules, its ability to regulate host lipid homeostasis suggests that fluctuations in its activity may modulate bile acid metabolism and host lipid metabolism enzyme expression, thereby indirectly participating in host fatty acid and phospholipid metabolic pathways. Additionally, *Prevotella copri* has been associated with increased inflammation, fat accumulation, and chronic inflammatory responses in adipose tissue (Chen et al., 2021). In mice, increased *Prevotella* in the gut and elevated serum phosphatidic acid levels exacerbate intestinal inflammation (Xiao et al., 2024). Therefore, the enrichment of *Prevotella* and elevated serum phosphatidic acid in infertile PCOS women may synergistically promote chronic inflammation.

This study also identified environmental endocrine disruptors (EDRs) such as deltamethrin and polystyrene sulfonate in serum metabolomic profiles. Low-dose deltamethrin exposure has been shown to increase *Prevotella* abundance in mouse intestines, whereas higher doses cause a significant decline in *Prevotella* (Fenech and Baron, 2025). Microplastics entering the gut can lead to dysbiosis and a reduction of beneficial bacteria (Thin et al., 2025). EDRs are exogenous substances that may disrupt host intestinal homeostasis by affecting the *Gemella* and *Prevotella* bacterial communities (Yong et al., 2020). When EDR exposure and dysbiosis coexist, they may produce synergistic harmful effects and aggravate intestinal and systemic inflammation (Giambò et al., 2022). More importantly, microbial dysbiosis may enhance the bioavailability of environmental pollutants in the body. A reciprocal relationship between environmental pollutants and microbial dysbiosis in infertility may exacerbate a cascade of detrimental effects, including endocrine disturbances, chronic inflammation, impaired uterine receptivity, and defective embryo implantation (Sun et al., 2025). Future longitudinal or intervention studies are needed to confirm this microbiota–metabolite–environment axis.

### 4.5 Innovations and limitations

This study has several strengths. It is the first multi-omics investigation to stratify PCOS patients by fertility status, moving beyond general PCOS-versus-healthy comparisons to address a critical clinical gap. By integrating deep phenotyping with 16S rRNA sequencing and untargeted metabolomics, we identified a cohesive microbiota–metabolite signature associated with infertility rather than isolated biomarkers. A machine learning model leveraging these features achieved promising diagnostic accuracy (AUC = 0.833) on an independent test set, underscoring its potential clinical translatability. Furthermore, the convergence of correlation analyses into plausible biological axes—such as the *Turicibacter*-Trichostachine association and the *Prevotella*-Phosphatidic acid link—provides a mechanistic framework for future research.

However, limitations include the cross-sectional design and modest sample size, which preclude causal inference and may limit the detection of subtle interactions. The predictive model requires external validation in larger cohorts. Methodologically, untargeted metabolomics findings—particularly for xenobiotics such as as deltamethrin—require targeted LC–MS/MS validation, and functional insights from PICRUSt2 remain predictive, needing confirmation via metagenomic sequencing or experimental models. Finally, reported microbiota–metabolite correlations do not imply causality. Longitudinal, interventional, and *in vitro* studies are needed to dissect the gut–ovary axis and validate these findings.

## 5 Conclusion

This study demonstrates that infertility in PCOS is associated with a distinct gut microbiota–metabolite profile, characterized by enrichment of Turicibacter, depletion of short-chain fatty acid–producing bacteria such as *Alistipes finegoldii* and *Burkholderiales* bacterium, and dysregulation of key serum metabolites, including elevated phosphatidic acid (22:1/20:3) and trichostachine. These alterations correlate with hyperandrogenism, abnormal gonadotropin levels, and enhanced inflammatory and xenobiotic stress responses, implying a synergistic role in the exacerbation of reproductive dysfunction. Although limited by its cross-sectional design and modest sample size, our integrated multi-omics approach provides novel insights into the microbiota–metabolite–-fertility axis in PCOS, highlighting potential mechanisms mediated by microbial–host co-metabolism and metabolic inflammation. Future studies involving larger cohorts and functional validations are warranted to establish causal relationships and explore therapeutic strategies targeting these pathways.

## Data Availability

The 16S rRNA sequencing data presented in this study are deposited in the NCBI Sequence Read Archive (SRA) repository under accession number PRJNA1348884. The raw metabolomics data have been deposited to the MetaboLights repository under accession number MTBLS14097.
