# Supplementary figures and images for: The gut microbiota–metabolite axis in polycystic ovary syndrome: differential characteristics between infertile and conceived populations
Source: Front Microbiol. 2026 Mar 27;17:1705096. doi: 10.3389/fmicb.2026.1705096 (PMC13067366; doi:10.3389/fmicb.2026.1705096)

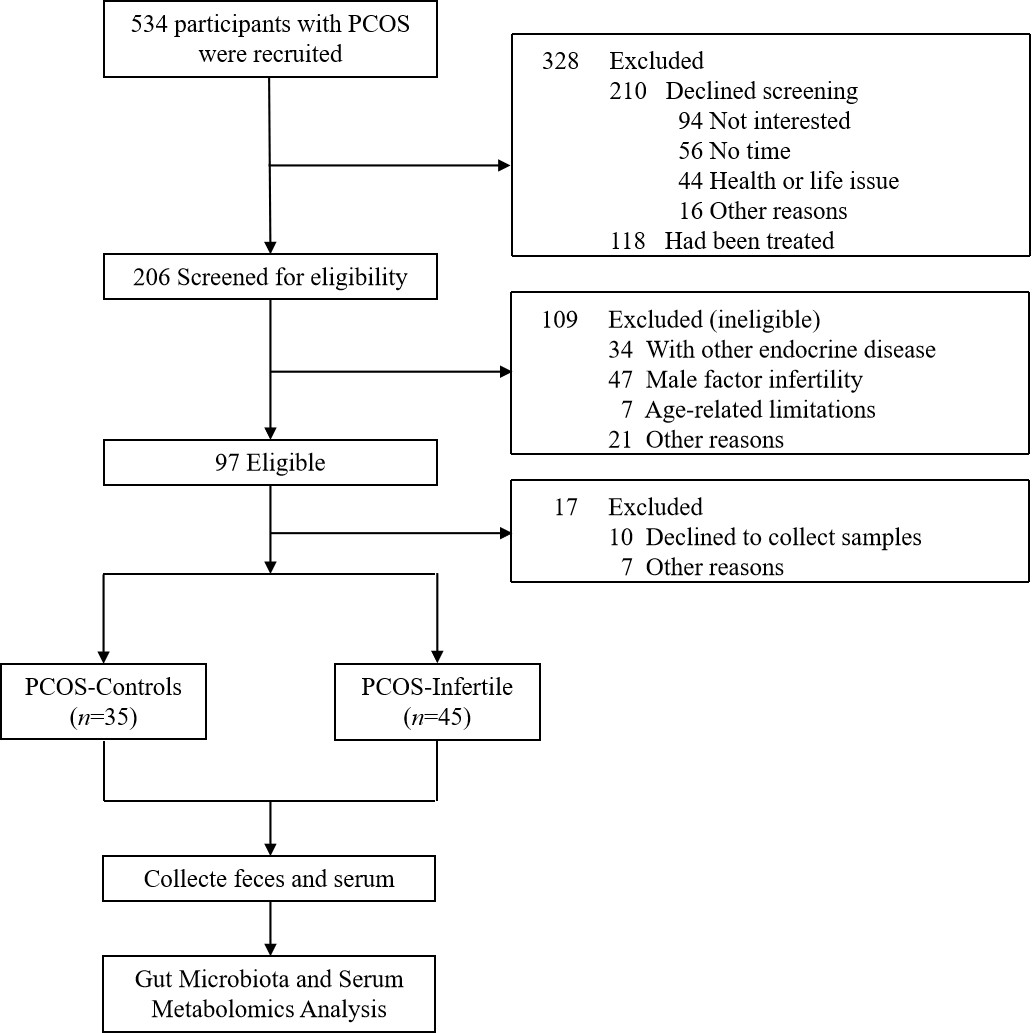

Supplement: Supplementary file 1 [file Image_1.jpeg]
